# Left Ventricular Systolic Impairment after Pediatric Cardiac Surgery Assessed by STE Analysis

**DOI:** 10.3390/healthcare9101338

**Published:** 2021-10-09

**Authors:** Massimiliano Cantinotti, Pietro Marchese, Marco Scalese, Paola Medino, Vivek Jani, Eliana Franchi, Pak Vitali, Giuseppe Santoro, Cecilia Viacava, Nadia Assanta, Shelby Kutty, Martin Koestenberger, Raffaele Giordano

**Affiliations:** 1 Fondazione G. Monasterio CNR-Regione Toscana, 54100 Massa, Italy; cantinotti@ftgm.it (M.C.); pitrino91@gmail.com (P.M.); dottpaooola@live.it (P.M.); eliana.franchi@ftgm.it (E.F.); pakv2001@yahoo.com (P.V.); giuseppe.santoro@ftgm.it (G.S.); cecilia.viacava@ftgm.it (C.V.); assanta@ftgm.it (N.A.); 2 Institute of Clinical Physiology, IFC, National Research Institute (CNR), 56121 Pisa, Italy; scalese@ifc.cnr.it; 3 Department of Pediatrics, University of Pisa, 56121 Pisa, Italy; 4 The Helen B. Taussig Heart Center, The Johns Hopkins Hospital and Johns Hopkins University, Baltimore, MD 21218, USA; vpjani@ucsd.edu (V.J.); shelby.kutty@gmail.com (S.K.); 5 Department of Pediatrics, Division of Pediatric Cardiology, Medical University, 8036 Graz, Austria; martin.koestenberger@medunigraz.at; 6 Adult and Pediatric Cardiac Surgery Unit, Department of Advanced Biomedical Sciences, University of Naples Federico II, 80138 Naples, Italy

**Keywords:** echocardiography, congenital heart disease, cardiac surgery, speckle tracking echocardiography

## Abstract

Background: Speckle-tracking echocardiography (STE) has gained increasing value in the evaluation of congenital heart diseases (CHD); however, its use in pediatric cardiac surgery is limited. Aim: To evaluate left ventricular (LV) systolic impairment after biventricular pediatric cardiac surgery by STE strain (ε) analysis. Methods: We prospectively enrolled 117 children undergoing cardiac surgery for CHD. Echocardiography was performed at four different times: pre-operatively, 12–36 h (Time 1), 3–5 days (Time 2), and 6–8 days (Time 3). Images were obtained in the 4-2-and 3 apical chamber’s views to derive LV global and regional (basal/mid/apical) ε values. Results: At different postoperative times, we performed 320 examinations in 117 children (mean age: 2.4 ± 3.9, range: 0–16 years); 117 age-matched healthy children served as controls. All global, basal, and mid LVε values decreased after surgery; the lowest values being at Time 1 (*p* < 0.0001), which increased thereafter. At discharge, all global, basal, and mid LVε values remained lower than in pre-operative and healthy children (*p* < 0.05). Instead, apical segments (lowest at baseline) increased after surgery (*p* < 0.0001) but remained lower compared to controls. LV ejection fraction (LVEF) decreased at Time 1 (*p* = 0.0004) but promptly recovered to Time 2 and normalized at Time 3. Conclusions: STE ε analysis revealed a significant LV systolic impairment after surgery with amelioration thereafter but incomplete normalization at discharge. Base-apex differences emerged with apical segments that, contrary to all the other regions, showed relative hypercontractility after surgery. The slower recovery of LVε values compared to LVEF suggests that STE ε analysis may be more accurate for the follow-up of mild LV post-surgical impairment.

## 1. Introduction

Speckle-tracking echocardiography (STE) has demonstrated significant value and an increasing impact in the evaluation of ventricular systolic function in children with congenital heart disease (CHD) [1]. STE is clinically feasible, and is more reproducible, in the assessment of left ventricular (LV) function, compared to LV ejection fraction (LVEF) [2,3]. STE strain analysis furthermore allows for a more accurate, fast, semi-automated assessment of regional ventricular function [2,3]. Despite its advantages, the application of STE for the evaluation of LV function in the perioperative period of pediatric cardiac surgery has so far been limited [4,5,6,7,8,9]. Few studies have been specifically designed to assess both the feasibility and postoperative left ventricular [4,5,6,7,8,9] and right [5,6,7,8] ventricular strain in CHD children after cardiac surgery. These studies, however, were conducted in small patient cohorts (<40 subjects) or had a retrospective study design [4,5,6,8]. Prospective studies on changes in ventricular systolic function as a function of postoperative time evaluated by STE strain analysis in pediatric cardiac surgery are currently lacking. Previous studies, furthermore, used small a sample size of healthy children (<80 subjects) [4,7] for comparison of postoperative strain values with ranges of normality, or did not perform comparisons with normal values at all [3,5,8,9]. The recent availability of nomograms for STE ventricular strain values, calculated in a large sample size (up to 721 children) covering all pediatric age groups (0–17 years) [10,11,12,13,14,15], allows for a more accurate comparison of strain values in children with CHD after surgery with an age-specific range of normality, thus enabling a better understanding of the degree of their alteration. Regional systolic function after pediatric cardiac surgery was further evaluated only in a small study (25 subjects) using 3D STE [9], while no data are reported for conventional 2D STE.

The aim of our current investigation was to evaluate left ventricular (LV) systolic impairment after pediatric biventricular cardiac surgery assessed by global and segmental longitudinal STE strain (ε) analysis.

## 2. Methods

From May 2017 to January 2021, children undergoing biventricular cardiac surgery under cardiopulmonary bypass (CPB) for CHD were prospectively enrolled at a Single Institution (Fondazione CNR-Regione Toscana, G. Monasterio, Massa, Italy). Echocardiographic examinations were performed pre-operatively and at three different postoperative times, namely 12–36 h (Time 1), 3–5 days (Time 2), and at 6–8 days (Time 3). In complicated patients, echocardiograms were repeated whenever required for alterations to treatment. No examinations other than those necessary for treatment were performed. Using a pool of 721 of healthy children collected for a previous study [10], a group of 117 age-matched normal subjects (mean 2.83 ± 4.89 years old) were used as controls.

Echocardiograms were performed using the Philips iE33 system (Philips Medical Systems, Bothell, WA, USA) with 8 MHz and 5 MHz transducers. All studies were performed with simultaneous electrocardiographic monitoring. Images were obtained in the apical four chambers view. Images were obtained in the apical four-chamber (4 Ch), three-chamber (3 Ch), and two-chamber (2 Ch) views.

STE-derived longitudinal strain was measured for 4 Ch, 3 Ch, and 2 Ch views using offline analysis by vendor-specific software (QLAb 10, Philips Medical Systems, Bothell, WA, USA). Global LV longitudinal strain (LVGL ε) and regional (basal, mid, and apical) ε values were automatically derived from all views measured with a standard six-segments model (e.g., 6 segments for each view, 18 segments globally) [16,17]. For the final analysis, single basal, mid, and apical segments values were summed, and a mean was obtained. Measurements were only made if unambiguous views were available on high quality images. Segments deemed inadequately tracked in one or more views were excluded from analysis.

Two independent experienced pediatric cardiologists (M.C., E.F.) acquired images and performed measurements. The low rate of inter- and intra-observer variability in ventricular ε has been described elsewhere [10]. Left ventricular ejection fraction was calculated by the biplane Simpson’s method in a semi-automated fashion with manual correction and was classified as decreased (<40%), mildly decreased (40–50%), and normal (>50%) for all subjects evaluated [18].

Exclusion criteria for this study included: age > 18 years, univentricular heart patients, and any need for postoperative circulatory assistance. Subjects without an examination 12–36 h postop (Time 1) were also excluded. Furthermore, only subjects in which ventricular ε analysis was deemed feasible for at least 80% of the parameters evaluated were included in this study. 

### 2.1. Ethics

Approval for this study was obtained from the Local Ethics Committee (Comitato Etico Meyer no. 62/2016). Parents or legal guardians of all the children were informed and provided written consent for participation in this study.

### 2.2. Surgical and Medical Management 

The preoperative anesthetic approach, intra-operative CPB strategy, and postoperative intensive care unit (ICU) management followed standard institutional practices as described in previously studies [19]. Non-iodinated topical antiseptics were used for every patient. A standard technique was used to institute CPB (roller pump, disposable membrane oxygenator, and arterial filter) and involved bicaval drainage and ascending aorta perfusion. Different myocardial protection approaches (anterograde cold crystalloid or blood cardioplegia) and degrees of body cooling were used ranging from 19° to 35 °C, depending on the surgical strategy. In the postoperative period, hemodynamic management consisted of epinephrine, milrinone, dopamine, noradrenaline, and Levosimendan. Intravascular volume expansion was utilized as needed and consisted of 20% human albumin or fresh–frozen plasma. Diuretic management usually involved furosemide (1–10 mg/kg/day).

The vasoactive–inotropic score (VISmax) was calculated as a weighted sum of all administered inotropes and vasoconstrictors, according to current standards [20,21]. Five VISmax categories: 0–5, >5–15, >15–30, >30–45, and >45 points, were calculated as previously indicated [20,21].

### 2.3. Statistical Analysis

All continuous variables and categorical variables were expressed as mean standard deviation (SD) and as number of cases and percentage, respectively. Comparison of continuous variables at different time points was performed using Wilcoxon test and nonparametric test for trends, as appropriate. Comparison of categorical variables at different time points was performed using a chi-square (Cochran-Armitage) test for trends in proportions. Comparison of age class was performed using a Mann–Whitney U test and a chi-square test as appropriate. Additionally, Pearson correlation coefficients (r) between strain values, operative data, and outcome parameters were determined. The control group of normal subjects was selected by 1:1 propensity score matching. We performed an exact match for age.

All calculations were performed using SPSS v23 (SPSS Inc., Chicago, IL, USA) and STATA v13 software. A *p* < 0.05 was considered significant.

## 3. Results

### 3.1. Population

Echocardiographic examinations with STE analysis were performed in 136 children after pediatric cardiac surgery. Of these studies, 19 children were excluded either for a lack of a sufficient number of examinations or for incomplete examinations. A total of 320 examinations were performed at different postoperative times in the remaining 117 children (median age: 6.48 months, IQR: 1.28–27.3 months) undergoing cardiac surgery for CHD; for each patient, a single evaluation before surgery was conducted. The most common CHD, such as septal or atrioventricular defects, tetralogy of Fallot or pulmonary atresia, transposition of the great vessels, and left ventricular tract obstruction, were treated using standard biventricular correction techniques using a cardiopulmonary bypass. Among these, 26 children were neonates (median age: 9 days; IQR: 6.5–11.5 days), 31 were infants (median age: 92 months; IQR: 45–139 days), and 60 were older children (median age: 1.92 years, IQR: 0.95–6.8 years). Additional demographic data are reported in Table 1.

### 3.2. Feasibility

Feasibility, as assessed by the total number of studies from which relevant ventricular ε parameters were derived, ranged from 85.3–97.8% for all parameters. Feasibility was observed as similar across all age groups (Table 2).

### 3.3. Preoperative LV Strain Values

LV global (4 Ch, 2 Ch, 3 Ch) and LVGL preoperative strain values were not different from healthy subjects. LV mid segmental strain values were also similar in CHD and in healthy (*p* = 0.14) subjects, while apical segments were lower (*p* < 0.0001) and basal values were higher (*p* < 0.0001) in CHD. These data are summarized in Table 3 and Table 4.

### 3.4. Progression of Global LV Strain Values as a Function of Postoperative Time

**Figure 1:**
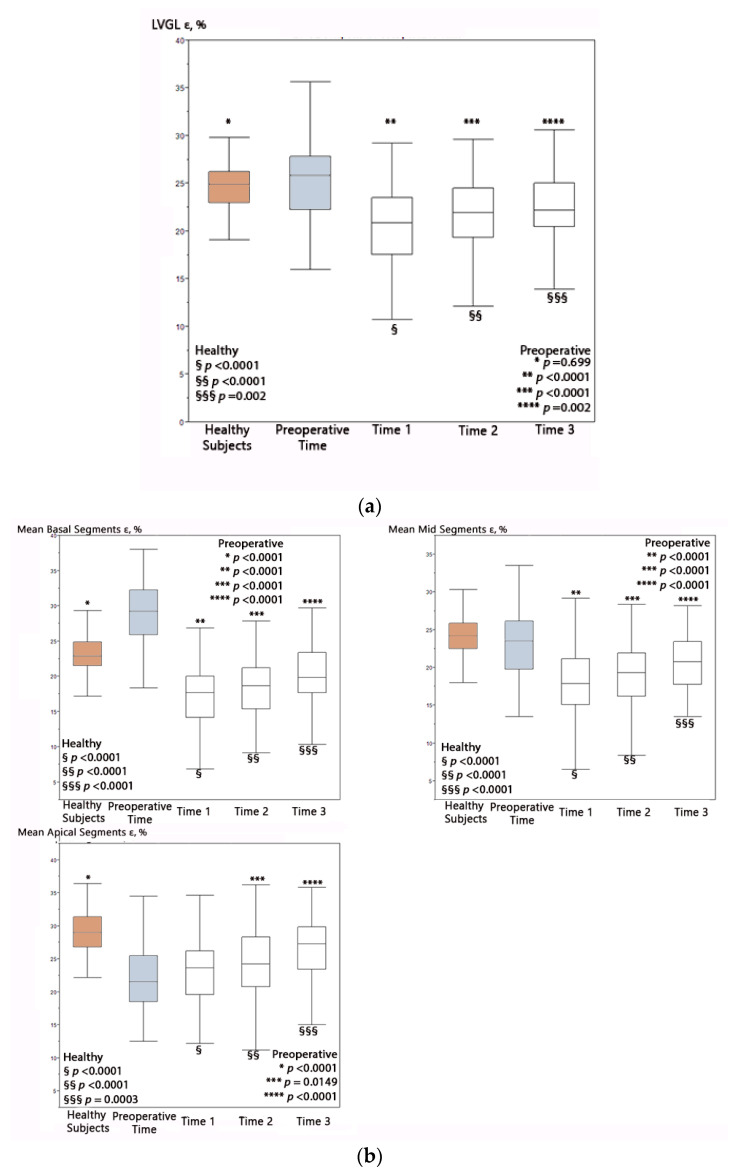
Median and interquartile range of global ventricular ε values over time in the overall population. On the left are the i values of the differences among healthy subjects (brown box) and different pre- and postoperative times, while on right, the i values of differences among preoperative time (gray box) and postoperative times and healthy subjects are shown: (**a**) global LV ventricular ε values; (**b**) segmental LV ventricular ε values. Bold horizontal line = median; box = interquartile range. LVGL = left ventricle global longitudinal strain.

Table 3 reports all postoperative times for 2 Ch, 3 Ch, 4 Ch, and LVGL ε postoperative times. All global LV ε values were lower at all postoperative times compared with preoperative (*p* all < 0.0001 for Time 1 and Time 2 and *p* ranging from 0.0002 to 0.048 for Time 3). The lowest LV ε were observed at 12–36 h postop (Time 1), with a significant trend to increase thereafter (*p* < 0.007). At Time 2, only LVGL ε values had a significant increase (*p* = 0.03) compared to Time 1. At Time 3, instead, all LV ε were higher than at Time 1 (*p* < 0.0001); however, they remained significantly lower compared to pre-operative values and controls (*p* ranging from < 0.0001 to 0.048). These results are summarized in Figure 1.

Analysis of LVGL ε in percentiles referenced from pediatric nomograms [10] yielded similar results. Before surgery, 14.8% had LVGL ε values below the 5th percentile, and in half of the subjects (50%) LVGL ε values were above the 50th percentile. At Time 1, a total 61.4% had LVGL ε values less than the 5th percentile and only 8.8% had LVGL ε values above the 50th percentile. Normalization was incomplete upon discharge. At Time 4, LVGL ε values were below the 5th percentile in 43.7%, and more than half of the subjects (52.1%) showed LVGL ε values between the 5th and 50th percentile (Table 5).

### 3.5. Progression of Segmental LV Strain Values as a Function of Postoperative Time

At Time 1 and Time 2, all segmental LV strain values were lower than preoperative time and controls (*p* ranging from <0.0001 to 0.01), except for apical regions, which showed values similar to baseline at Time 1 and even higher values than pre-operatively at Time 2 (*p* = 0.0149) and Time 3 (*p* < 0.0001) (Table 3).

From Time 1 to Time 2, no segment reported a significant improvement, while at Time 3, all segments were significantly higher than at Time 1 (*p* all< 0.0001), but basal and mid segments were still lower compared to preoperative data (*p* = 0.0001, and *p* = 0.004, respectively), while apical segments were higher than baseline (*p* < 0.0001). At Time 3, all the segmental strain values (including apical segments) remained lower than in healthy controls (*p* varying from< 0.0001 to 0.0003). These data are summarized in Table 4.

### 3.6. Differences of LV Ventricular Strain Values among Age Groups 

At various postoperative times, neonates tended to have higher mean LV ε values compared to other age groups; however, differences were not significant, with limited exceptions (Table A1).

### 3.7. Strain and Left Ventricular Ejection Fraction

LV ejection fraction demonstrated a strong correlation with all LV strain parameters, namely 2 Ch LV ε (beta = 0.383; *p* < 0.001), 3 Ch LV ε (beta = 0.37; *p* < 0.001), 4 Ch LV ε (beta = 0.34; *p* < 0.001), and LVGL ε (beta = 0.36; *p* < 0.001), as depicted in Table A2.

LV ejection fraction demonstrated a similar trend in improvement as a function of postoperative time as LV ε values. Compared to LV strain, however, the degree of impairment of LVEF was milder, and the recovery was faster. The LVEF at Time 1 was significantly lower compared to preoperative time and to Time 2 and Time 3 (*p* varying from 0.0009 to 0.031); however, at Time 2 and Time 3, no significant differences in LVEF from baseline were depicted (*p* 0.17 and *p* 0.9, respectively). Briefly, at baseline, the totality of patients had a normal LVEF (>50%); at Time 1, 4.42% of subjects had LVEF < 40%; 12.39% of subjects had an LVEF between 40–50%. At Time 3, no patients had an LVEF < 40%, while 3.17% of patients had an LVEF between 40 and 50%. These results are summarized in Table 6.

### 3.8. Inotrope Data and Vasoactive–Inotropic Score 

Data on inotropes and vasoactive–inotropic score are reported in Table A3. LVGL ε at Time 1 was higher in children receiving inotropes (*p* < 0.03). No other significant differences in LV ε values were reported between patients receiving or not receiving inotropes, either at Time 1 or at Time 2, as shown in Table A4. At univariate analysis, no correlations were reported between global and segmental LV STE ε values and the vasoactive–inotropic score, either at Time 1 or at Time 2.

### 3.9. Correlation of Ventricular Strain with Conventional Risk Factors and Operative Data

LVGL ε demonstrated a significant, albeit weak, correlation with cardiopulmonary bypass time (CPB) (beta = −0.02, *p* = 0.0007), cross clamp time (beta = −0.0222, *p* = 0.007), age (beta = −0.287; *p* < 0.001), and BSA (beta = −0.027; *p* < 0.001). All ε values and segmental ε values, with mid segments the only exception, were inversely correlated with age (*p* ranging from <0.0001 to 0.03) and body surface area (*p* ranging from <0.0001 to 0.014). Aristotle Score and STAT score were not related with ε values and segmental ε values, whilst a negative correlation was found between cardiopulmonary bypass time and LV 4 Ch ε, LV 3 Ch ε, LV GL ε, and mid segments (*p* ranging from 0.0006 to 0.04), as summarized in Table A2.

## 4. Discussion

We prospectively evaluated LV systolic impairment in children undergoing biventricular cardiac surgery for different CHDs by STE strain analysis. There are limited data on LV STE application in pediatric cardiac surgery, deriving either from small sample sizes (<40 subjects) or retrospectively designed studies [4,6,8,9]. These studies either used small sample sizes of healthy children for comparison of strain data in CHDs with normal ranges [4,7] or did not perform a comparison with normality at all [3,5,8,9]. Regional deformation has also been poorly evaluated so far [9]. Our results tend to reinforce previous observations (4–9) and provide novel findings that cover some gaps as yet unclosed by previous studies [4,5,6,7,8,9]. First, this is one of the first studies to analyze both global and regional LV systolic impairment after pediatric cardiac surgery assessed by STE strain analysis. Second, for a better understanding of the degree of LV impairment after surgery, we compared post-surgical STE strain values to those of age-matched controls, as derived from a large population of healthy children [10]. Post-surgical global LV strain values were expressed both as absolute values and as percentiles referenced from pediatric nomograms [10], with the latter providing a semi-quantitative assessment of the degree of LV systolic impairment. Third, we evaluated LVEF by the biplane Simpson’s method, a basic parameter whose data are surprisingly limited in children after cardiac surgery [22,23,24].

Regarding feasibility, our data confirm that STE-derived ventricular strain analysis is highly feasible in a wide range of CHDs across different pediatric age groups. We reported a feasibility of 85–98%, which is consistent with that presented in previous studies (87–93% feasibility) [4,5]. The observed postoperative time trend of global LV strain values in our cohort is in line with previous observations [3,4,5,6,9]. Postoperatively, all LV strain parameters were constantly and significantly decreased compared to preoperative values and compared to normal pediatric subjects [10], either when expressed as absolute values or in percentiles. As expected, the lowest ventricular strain values were observed at the first postoperative time point, between 12 and 36 h after surgery, with progressive recovery thereafter [6,8]. Nonetheless, upon discharge, ventricular strain remained impaired compared to pre-operative values and to normal pediatric subjects. These results are consistent with those observed in a retrospective study [6] of 204 children (age: 3.7 ± 5.1 years) undergoing biventricular surgery for different CHDs, where GLVS values at discharge were significantly decreased compared to pre-operative values and to those of 78 healthy controls (age: 3.6 ± 5.0 years).

Analysis of regional strain values showed similar time trends in global strain values, but significant differences in the base-apex emerged. Basal and mid segment values decreased after surgery, while values of the apical regions (who had the lowest values pre-operatively) increased. In a small study [9] of 25 children (age 9.4 ± 9.8 years), evaluating 3D LV ventricular strain at 1 week and 1 month after cardiac surgery for CHD, no significant differences in regional deformation were noted, except for a more pronounced reduction in strain values in the LV-free wall middle segments for cyanotic patients (*p* = 0.037). Curiously, the trend in postoperative apical hypercontractility we described herein is opposite to the one previously reported in adult [25,26] patients. In 182 patients, following different types of cardiac surgery (e.g., aortic valve replacement, mitral valve repair or replacement, coronary artery bypass graft), segmental function evaluated by 3D strain was greatly impaired in apical when compared to basal segments. In this study [25], however, many patients had left anterior descending coronary artery disease; thus, the differences in regional strain may be attributed to pre-existing coronary artery disease. The basal and middle segment strain values, in fact, seem to be ameliorated after coronary artery bypass surgery due to revascularization [25,26]. Thus, a comparison of adult data with those of children who usually have a normal coronary artery bed is difficult and should be avoided. We may speculate that, in pediatric cardiac surgery, apical segments are usually not involved by surgical maneuvers, while basal and middle segments are often involved or close to surgical sutures.

LVEF had a strong correlation with all LV strain parameters. Furthermore, LVEF improved with increasing postoperative time; however, the LVEF was observed to be less impaired and demonstrated faster recovery compared to strain values. In our study, 16.8% of subjects had an impaired LVEF observed 24–36 h postoperatively vs. 61.4% with LVGL strain values < 5th percentile and 29.4% < 25th percentile. At hospital discharge, only 3.17% of children had mild LVEF dysfunction while up to 43.7% had LVGL strain values < 5th percentile and 18.7% < 25th percentile. Surprisingly, limited data exist regarding postoperative ventricular function after pediatric cardiac surgery [22,23,24]. In these studies, different methods were used to assess ventricular function, namely EF by area length [20,21] and M-mode fractional shortening [22,23,24]. To the best of our knowledge, there are no well-defined criteria to classify ventricular function impairment in children. As a result, we utilized adult criteria for our study [18]. The degree of the decrease in ventricular strain was observed to be mildly correlated with surgical risk as assessed by age, BSA, and duration on CPB. These data are consistent with those previously reported [4,6,9]. In a study of 33 pediatric subjects (mean age: 4.2 ± 2.4 years.), Perdreau et al. [4] showed that LV longitudinal strain moderately correlated with aortic cross clamp duration on postoperative day 0 (*r* = 0.47, *p* = 0.016) and postoperative day 1 (*r* = 0.53, *p* = 0.010) [1]. However, no significant association among LV strain values and CPB [6,9] were reported by other authors. 

From a clinical point of view, STE ventricular longitudinal strain analysis is easy and fast, requiring the same time for imaging acquisition and subsequent data analysis as for the calculation of LVEF, and offering the advantage of being a more precise assessment of regional function. The higher degree of impairments of strain values and the slower recovery of LV strain values suggest that STE analysis may reveal even mild systolic impairment, which is often not diagnosed with conventional echocardiographic markers. Thus, LV STE strain analysis may be used for the better quantification and follow-up of global and regional LV systolic impairments after pediatric cardiac surgery, and it may guide progressive therapy reduction and suspension.

### Limitations

A limitation of our work is the use of vendor-specific software for the evaluation of ventricular strain parameters from STE. Our study did not evaluate the radial or circumferential strain, as well as strain rate, but was limited to ventricular longitudinal strain, which is currently the only parameter recommended in adult guidelines [16,17]. We used percentiles only for global ventricular strain values [10], since, for regional indexes, normal values are still limited. Another limitation was the lack of a standardized timing for echocardiographic examination. The timing of the first examination varied between 12 and 36 post-surgeries, depending on clinical indication. Furthermore, not all patients had a complete examination at all time points. Data of follow-up are also lacking. Preoperative LV strain values should be evaluated with caution, since we retain that, in uncorrected CHD, the calculation of ventricular strain is limited due to the presence of significant shunts and by alterations in ventricular geometry. Subjects in our investigation were to some extent heterogeneous and displayed a wide variety of cardiac defects across different age groups.

## 5. Conclusions

We describe LV systolic impairment after pediatric cardiac surgery assessed by STE global and segmental strain analysis. Our data showed that LV systolic function was significantly impaired after pediatric cardiac surgery, as demonstrated by the significant decrease in global and segmental LV strain parameters compared to preoperative and healthy subjects. The lowest LV strain values were observed at Time 1, with progressive recovery during postoperative days but without complete normalization at discharge. We found a variability in the LV base-to-apex strain response with apical regions that, contrary to basal and mid segments, increased after surgery. LVEF values showed strong correlations and similar postoperative time trends to LV strain values, but a milder degree of impairment and a faster recovery. Thus, our data suggest that STE ventricular strain analysis may reveal subclinical myocardial injury not observed with conventional echocardiographic markers such as LVEF. LV STE analysis may be particularly helpful for the follow-up of regional and mild global post-surgical LV systolic impairments and recovery after cardioplegia; these are not always visible with conventional echocardiographic markers. Thus, this approach might guide therapy reduction and suspension. Further studies with larger cohorts are required to validate and reinforce our findings.

## Figures and Tables

**Table 1:** Population.

Characteristic	Neonates		Infant		Older	
Variables	Mean *(Median)*	Dev std *(IQR)*	Mean *(Median)*	Dev std *(IQR)*	Mean *(Median)*	Dev std *(IQR)*
Age (years)	0.027 (0.02)	0.01 (0.02–0.03)	0.25 (0.25)	0.13 (0.12–0.38)	4.37 (1.92)	4.63 (0.95–6.8)
Age (months)	0.33 (0.3)	0.19 (0.22–0.38)	3.12 (3.07)	1.57 (1.5–4.63)	53.16 (23.33)	56.29 (11.5–82.76)
Age (days)	9.88 (9)	5.58 (6.5–11.5)	93.74 (92)	46.95 (45–139)	1594.89 (700)	1688.57 (345–248.75)
Weight (Kg)	3.25 (3.29)	0.62 (2.98–3.57)	5.03 (4.92)	1.64 (3.95–5.93)	17.27 (11.03)	14.18 (6.75–24)
Height (cm)	49.92 (51)	5.26 (48.5–52.5)	60.47 (59)	10.50 (53.5–64.25)	95.12 (83)	34.61 (67–124)
BSA (m2)	0.21 (0.21)	0.03 (0.21–0.23)	0.29 (0.29)	0.06 (0.25–0.35)	0.66 (0.5)	0.38 (0.36–0.9)
CPB (min)	142.74 (177)	103.13 (0–219)	102.48 (93)	63.68 (63–134)	101.74 (99.5)	47.51 (69.5–120)
Cross Clamp (min)	93.38 (99.5)	70.62 (27–128.75)	62.00 (62)	43.11 (36.5–87)	65.36 (61.5)	35.94 (40–85)
Aristotle score	8.77 (10)	1.95 (6–10)	6.89 (6.3)	1.37 (6–8)	6.38 (6.3)	2.33 (6–8)
STAT	2.11 (3)	0.99 (1–3)	1.46 (1)	0.81 (1–2)	1.45 (1)	0.67 (1–2)
**Classification**	**Neonates**		**Infant**		**Older**	
LVVO	1.00		16.00		16.00	
RVPO	0.00		5.00		16.00	
LVPO	8.00		2.00		10.00	
TGA	16.00		3.00		0.00	
RVVO	0.00		1.00		14.00	
AVSD	1.00		2.00		4.00	
Other	0.00		2.00		0.00	

Legend: AVSD = atrio-ventricular septal defects, BSA = body surface area, CPB = cardiopulmonary bypass, CHDS = congenital heart diseases, Dev Std = standard deviation, IQR = interquartile range, LVPO= left ventricle pressure overload CHDs (aortic stenosis, aortic coarctation, etc.), LVVO = left ventricle volume overload CHDs (ventricular septal defects, aorto-pulmonary windows, etc.), *n* = number, RVPO = right ventricle pressure overload CHDs (tetralogy of Fallot, pulmonary stenosis, etc.), RVVO = right ventricle volume overload CHDs (atrial septal defects, anomalous pulmonary venous return, etc.), TGA = transposition of the great arteries.

**Table 2:** Feasibility at different age groups.

STE	Neonates (%)	Infants (%)	Older (%)	Total (%)
***LV 4 Ch ε***	98.5	97.4	96.2	97.1
***LV 2 Ch ε***	96.5	94.8	96.2	96.3
***LV 3 Ch ε***	97.5	94.8	96.2	96.3
***LVGL ε***	98.5	97.4	97.7	97.8

Legend: LV = left ventricle, 4 Ch = left ventricle four-chamber view, 2 Ch = two-chamber view, 3 Ch = three-chamber view, LVGL = left ventricle global longitudinal strain, STE = Speckle Tracking Echocardiography.

**Table 3:** Mean and standard deviation of LV ε values at different time evaluations.

STE	Before Surgery (Pre)		Time 1		Time 2		Time 3		Healthy Subjects	
Variables	Mean	Dev std	Mean	Dev std	Mean	Dev std	Mean	Dev std	Mean	Dev std
**LV 4 Ch ε**	24.98	4.22	20.81	4.91	21.76	4.49	23.4	4.49	24.93	2.77
**LV 2 Ch ε**	25.24	4.24	21.04	5.19	22.11	4.44	22.82	4.32	25.19	2.74
**LV 3 Ch ε**	25.46	4.24	20.17	4.8	20.93	4.43	22.61	3.91	23.8	3.48
**LVGL ε**	25.23	4.23	20.37	4.71	21.56	4.14	22.9	3.73	24.84	2.67
**Mean Basal Segments**	28.64	4.9	17.39	4.34	18.15	3.87	20.2	3.97	23.03	2.77
**Mean Mid Segments**	23.19	5.05	18	4.57	18.88	4.4	20.81	3.85	24.11	2.85
**Mean Apical Segments**	21.94	4.83	22.83	5.82	24.12	5.33	26.63	5.38	29.05	3.25

Legend: LV = left ventricle, 4 Ch = left ventricle four-chamber view, 2 Ch = two-chamber view, 3 Ch = three-chamber view, LVGL = left ventricle global longitudinal strain, STE = Speckle Tracking Echocardiography.

**Table 4:** Differences among mean and standard deviation of LV global ε values and segmental ε values at different time evaluations and comparison with healthy subjects.

STE	Pre vs. T1	Pre vs. T2	Pre vs. T3	Pre vs. Controls	Control vs. Time 1	Control vs. Time 2	Control vs. Time 3	Time 1 vs. Time 2	Time 2 vs. Time 3	Time 1 vs. Time 3
Variables	*p*	*p*	*P*	*p*	*p*	*p*	*p*	*p*	*p*	*p*
LV 4 Ch ε	<0.0001 *	<0.0001 *	0.048 *	0.9	0.0009 *	0.0077 *	0.005 *	0.6	0.5	0.23
LV 2 Ch ε	<0.0001 *	<0.0001 *	0.003 *	0.9	<0.0001 *	<0.0001 *	0.0004 *	0.07	0.3	0.007 *
LV 3 Ch ε	<0.0001 *	<0.0001 *	0.0002 *	0.0084 *	<0.0001 *	<0.0001 *	0.07	0.2	0.01 *	0.0002 *
LVGL ε	<0.0001 *	<0.0001 *	0.002 *	0.47	<0.0001 *	<0.0001 *	0.002 *	0.03 *	0.03 *	<0.0001 *
Mean Basal Segments	<0.0001 *	<0.0001 *	<0.0001 *	<0.0001 *	<0.0001 *	<0.0001 *	<0.0001 *	0.19	0.0013 *	<0.0001 *
Mean Mid Segments	<0.0001 *	<0.0001 *	0.0042 *	0.14	<0.0001 *	<0.0001 *	<0.0001 *	0.16	0.005 *	<0.0001 *
Mean Apical Segments	0.33	0.0149 *	<0.0001 *	<0.0001 *	<0.0001 *	<0.0001 *	0.0003 *	0.09	0.004 *	<0.0001 *

Legend: LV = left ventricle, 4 Ch = left ventricle four-chamber view, 2 Ch = two-chamber view, 3 Ch = three-chamber view, LVGL = left ventricle global longitudinal strain, STE = Speckle Tracking Echocardiography. *: statistically significant.

**Table 5:** LVGL ε values distribution as percentiles at different time evaluations.

Percentile	<5th	5–10th	10–25th	25–50th	50–75th	75–90th	90–95th	>95th
**Preoperative**	14.8%	5.6%	7.4%	22.2%	16.7%	13.0%	5.6%	14.7%
**Time 1**	61.40%	5.26%	14.04%	9.65%	3.51%	5.26%	0.00%	0.88%
**Time 2**	53.26%	2.17%	17.39%	15.22%	6.52%	4.35%	1.09%	0.00%
**Time 3**	43.75%	4.69%	14.06%	18.75%	6.25%	7.81%	0.00%	4.69%

LVGL = left ventricle global longitudinal.

**Table 6:** Time trend of postoperative LV ejection fraction values.

Age Group	Time of Examination	Ejection Fraction (%)					
		Normal (>50%)		Mildly Decreased (40–50%)	Reduced (<40%)		
Newborn	Before Surgery	100.00%		0.00%	0.00%		
	Time 1	92.31%		7.69%	0.00%		
	Time 2	100.00%		0.00%	0.00%		
	Time 3	100.00%		0.00%	0.00%		
Infant	Before Surgery	100.00%		0.00%	0.00%		
	Time 1	78.57%		14.29%	7.14%		
	Time 2	85.71%		4.76%	9.52%		
	Time 3	100.00%		0.00%	0.00%		
Older	Before Surgery	100.00%		0.00%	0.00%		
	Time 1	81.36%		13.56%	5.08%		
	Time 2	82.61%		10.87%	6.52%		
	Time 3	93.10%		6.90%	0.00%		
All	Before Surgery	100.00%		0.00%	0.00%		
	Time 1	83.2%		12.39%	4.42%		
	Time 2	87.78%		6.67%	5.56%		
	Time 3	96.83%		3.17%	0.00%		
Ejection Fraction over time							
Ejection Fraction (%)	Mean ± dev std	63.7 ± 5.1		58.6 ± 9.7	61.6 ± 10.7		63.8 ± 7.9

Ejection Fraction		Before Surgery to Time 1	Before Surgery to Time 2	Before Surgery to Time 3	Time 1 to Time 2	Time 2 to Time 3	Time 1 to Time 3
Ejection Fraction (%)	*p*	0.0004 *	0.17	0.9	0.041 *	0.17	0.0004 *

*: statistically significant.

## Appendix A

**Table A1:** Mean and standard deviation of LV STE ε values at different time evaluations in different age groups.

Postoperative Times	STE	Neonates		Infant		Older		Older vs. Infant	Older vs. Neonates	Neonates vs. Infant
Variables	STE	Mean	Dev std	Mean	Dev std	Mean	Dev std	*p*	*p*	*p*
Time 1	LV 4 Ch ε	22.37	4.03	20.74	5.53	20.14	4.84	0.6	0.048 *	0.21
	LV 2 Ch ε	22.54	3.85	21.93	6.63	19.91	4.72	0.09	0.03 *	0.66
	LV 3 Ch ε	21.62	3.82	20.61	5.35	19.28	4.81	0.23	0.04 *	0.44
	LVGL ε	22.11	3.36	20.15	5.92	19.71	4.4	0.7	0.03 *	0.12
Time 2	LV 4 Ch ε	24.54	3.44	21.39	4.53	20.58	4.41	0.5	0.0004 *	0.013 *
	LV 2 Ch ε	24.96	3.79	21.84	3.43	20.84	4.59	0.3	0.0002 *	0.013 *
	LV 3 Ch ε	23.02	3.6	21	4.59	19.87	4.43	0.31	0.0048 *	0.11
	LVGL ε	24.22	3.25	21.3	3.68	20.38	4.21	0.36	0.0002 *	0.013 *
Time 3	LV 4 Ch ε	25.47	3.96	22.56	3.84	22.73	4.88	0.89	0.04 *	0.05
	LV 2 Ch ε	24.65	4.3	22.78	3.78	21.8	4.42	0.43	0.03 *	0.2
	LV 3 Ch ε	23.95	3.34	22.9	3.71	21.69	4.18	0.3	0.06	0.42
	LVGL ε	24.66	3.29	22.68	3.42	22.02	3.9	0.54	0.02 *	0.11

LV = left ventricle, 4 Ch = left ventricle four-chamber view, 2 Ch = two-chamber view, 3 Ch = three-chamber view, LVGL = left ventricle global longitudinal strain, STE = Speckle Tracking Echocardiography. For the simplicity of the reader, ε are expressed as positive numbers in all the tables. *: statistically significant.

**Table A2:** Correlation among LV STE ε values with age, BSA, surgical risk scores and parameters, and LVEF.

STE	Age		BSA		Aristotle Score	STAT	CPB		Cross Clamp		LVEF	
Variables	beta	*p*	beta	*p*	*p*	*p*	beta	*p*	beta	*p*	beta	*p*
LV 4 Ch ε	−0.4	0.0012 *	−4.8	0.0002 *	0.6	0.8	−0.01	0.04 *		0.2	1.34	<0.0001
LV 2 Ch ε	−0.5	<0.0001 *	−5.51	<0.0001 *	0.7	0.9		0.08		0.2	1.29	<0.0001
LV 3 Ch ε	−0.5	<0.0001 *	−5.47	<0.0001 *	0.7	0.7	−0.02	0.006 *		0.05	1.49	<0.0001
LVGL ε	−0.5	0.0002 *	−4.91	<0.0001	0.9	0.9	−0.02	0.0006 *	−0.03	0.007 *	1.51	<0.0001
Mean Basal Segments	−0.3	0.0015 *	−3.77	0.0013 *	0.5	0.7		0.13		0.4	1.04	<0.0001
Mean Mid Segments	−0.2	0.05	−2.31	0.06	0.8	0.05	−0.02	0.03 *		0.1	0.9	<0.0001
Mean Apical Segments	−0.6	<0.0001 *	−6.94	<0.0001 *	0.2	0.9		0.1		0.3	0.93	<0.0001

Legend: LV= left ventricle, EF = ejection fraction, 4 Ch = left ventricle four-chamber view, 2 Ch = two-chamber view, 3 Ch = three-chamber view, LVGL= left ventricle global longitudinal strain, STE = Speckle Tracking Echocardiography. For the simplicity of the reader ε are expressed as positive numbers in all tables. *: statistically significant.

**Table A3:** Categorical distribution of vasoactive or inotropes drugs at Time 1.

VISmax Category	Neonates		Infant		Older		All	
	%	*N*	%	*N*	%	*N*	%	*N*
0−5	42.31%	11	61.29%	19	73.33%	44	63.25%	74
5−15	46.15%	12	38.71%	12	25%	15	33.30%	39
15−30	11.54%	3	0.0%	0	1.67%	1	3.42%	4
30−45	0.0%	0	0.0%	0	0.0%	0	0.0%	0
>45	0.0%	0	0.0%	0	0.0%	0	0.0%	0
No Vasoactive–Inotropes Administration	11.50%	3	12.90%	4	45%	27	29.10%	34

VIS = vasoactive–inotropic score.

**Table A4:** Differences in LV ε values among patients whose were administered inotropes and patients whose did not.

STE	Inotropes-Group		No Inotropes-Group		Inotropes-Group		No Inotropes-Group		Comparison Inotropes vs. No Inotropes	
STE	Time 1				Time 2				Time 1	Time 2
Variables	Mean	Dev std	Mean	Dev std	Mean	Dev std	Mean	Dev std	*p*	*p*
LV 4 Ch ε	21.15	4.83	19.91	5.08	22.25	4.47	21.25	4.5	0.23	0.29
LV 2 Ch ε	21.6	5.06	19.58	5.31	22.57	4.15	21.63	4.72	0.07	0.31
LV 3 Ch ε	20.69	4.46	18.82	5.43	21.13	4.27	20.72	4.62	0.07	0.66
LVGL ε	21	4.07	18.84	5.81	21.95	3.99	21.15	4.29	0.03 *	0.35
Mean Basal Segments	17.75	3.94	16.53	5.16	18.07	3.46	18.22	4.29	0.17	0.85
Mean Mid Segments	17.93	4.1	18.17	5.64	18.84	4.11	18.91	4.73	0.8	0.9
Mean Apical Segments	23.3	5.14	21.67	7.16	24.74	5.04	23.48	5.59	0.17	0.26

Legend to Table. LV = left ventricle, 4 Ch = left ventricle four-chamber view, 2 Ch = two-chamber view, 3 Ch = three-chamber view, LVGL = left ventricle global longitudinal strain, STE = speckle tracking echocardiography. *: statistically significant.

## References

[1] Karlsen S., Dahlslett T., Grenne B., Sjøli B., Smiseth O., Edvardsen T., Brunvand H. (2019). Global longitudinal strain is a more reproducible measure of left ventricular function than ejection fraction regardless of echocardiographic training. Cardiovasc. Ultrasound.

[2] Klaeboe L.G., Edvardsen T. (2019). Echocardiographic assessment of left ventricular systolic function. J. Echocardiogr..

[3] Perdreau E., Séguéla P.-E., Jalal Z., Perdreau A., Mouton J.-B., Nelson-Veniard M., Guillet E., Iriart X., Ouattara A., Roubertie F. (2016). Postoperative assessment of left ventricular function by two-dimensional strain (speckle tracking) after paediatric cardiac surgery. Arch. Cardiovasc. Dis..

[4] Colquitt J.L., Pignatelli R.H. (2016). Strain imaging: The emergence of speckle tracking echocardiography into clinical pediatric cardiology. Congenit. Heart Dis..

[5] Pletzer S.A., Atz A.M., Chowdhury S.M. (2019). The relationship between pre-operative left ventricular longitudinal strain and post-operative length of stay in patients undergoing arterial switch operation is age dependent. Pediatr. Cardiol..

[6] de Boer J.M., Kuipers I.M., Klitsie L.M., Blom N.A., ten Harkel A.D.J. (2017). Decreased biventricular longitudinal strain shortly after congenital heart defect surgery. Echocardiography.

[7] Li Y., Wang X., Lv Q., Wang J., Yang Y., He L., Yuan L., Zhang L., Xie M. (2016). Impact of surgical correction of tetralogy of Fallot on short-term right and left ventricular function as determined by 2-dimensional speckle tracking echocardiography. Medicine.

[8] Karsenty C., Hadeed K., Dulac Y., Semet F., Alacoque X., Breinig S., Leobon B., Acar P., Hascoet S. (2017). Two-dimensional right ventricular strain by speckle tracking for assessment of longitudinal right ventricular function after paediatric congenital heart disease surgery. Arch. Cardiovasc. Dis..

[9] Rafieyian S., Roodpeyma S., Vahidshahi K., Moghadasi A. (2018). Evaluation of regional myocardial function by strain and strain rate before and after surgical repair of congenital heart anomalies. J. Tehran. Heart Cent..

[10] Cantinotti M., Scalese M., Giordano R., Franchi E., Assanta N., Marotta M., Viacava C., Molinaro S., Iervasi G., Santoro G. (2018). Normative data for left and right ventricular systolic strain in healthy Caucasian Italian children by two-dimensional speckle-tracking echocardiography. J. Am. Soc. Echocardiogr..

[11] Lorch S.M., Ludomirsky A., Singh G.K. (2008). Maturational and growth-related changes in left ventricular longitudinal strain and strain rate measured by two-dimensional speckle tracking echocardiography in healthy pediatric population. J. Am. Soc. Echocardiogr..

[12] Marcus K.A., Mavinkurve-Groothuis A.M.C., Barends M., van Dijk A., Feuth T., de Korte C., Kapusta L. (2011). Reference values for myocardial two-dimensional strain echocardiography in a healthy pediatric and young adult cohort. J. Am. Soc. Echocardiogr..

[13] Zhang L., Gao J., Xie M., Yin P., Liu W., Li Y., Klas B., Sun J., Balluz R., Ge S. (2013). Left ventricular three-dimensional global systolic strain by real-time three-dimensional speckle-tracking in children: Feasibility, reproducibility, maturational changes, and normal ranges. J. Am. Soc. Echocardiogr..

[14] Levy P.T., Mejia A.A.S., Machefsky A., Fowler S., Holland M.R., Singh G.K. (2014). Normal ranges of right ventricular systolic and diastolic strain measures in children: A systematic review and meta-analysis. J. Am. Soc. Echocardiogr..

[15] Kutty S., Padiyath A., Li L., Peng Q., Rangamani S., Schuster A., Danford D.A. (2013). Functional maturation of left and right atrial systolic and diastolic performance in infants, children, and adolescents. J. Am. Soc. Echocardiogr..

[16] Voigt J.-U., Pedrizzetti G., Lysyansky P., Marwick T.H., Houle H., Baumann R., Pedri S., Ito Y., Abe Y., Metz S. (2014). Definitions for a common standard for 2D speckle tracking echocardiography: Consensus document of the EACVI/ASE/Industry Task Force to standardize deformation imaging. Eur. Heart J. Cardiovasc. Imaging.

[17] Lang R.M., Badano L.P., Mor-Avi V., Afilalo J., Armstrong A., Ernande L., Flachskampf F.A., Foster E., Goldstein S.A., Kuznetsova T. (2015). Recommendations for cardiac chamber quantification by echocardiography in adults: An update from the American Society of Echocardiography and the European Association of Cardiovascular Imaging. Eur. Heart J. Cardiovasc. Imaging.

[18] Ponikowski P., Voors A.A., Anker S.D., Bueno H., Cleland J.G.F., Coats A.J.S., Falk V., González-Juanatey J.R., Harjola V.-P., Jankowska E.A. (2016). 2016 ESC Guidelines for the diagnosis and treatment of acute and chronic heart failure: The Task Force for the diagnosis and treatment of acute and chronic heart failure of the European Society of Cardiology (ESC)Developed with the special contribution of the Heart Failure Association (HFA) of the ESC. Eur. Heart J..

[19] Cantinotti M., Giordano R., Scalese M., Molinaro S., Della Pina F., Storti S., Arcieri L., Murzi B., Marotta M., Pak V. (2015). Prognostic role of BNP in children undergoing surgery for congenital heart disease: Analysis of prediction models incorporating standard risk factors. Clin. Chem. Lab. Med..

[20] Koponen T., Karttunen J., Musualowicz T., Pietiläinen L., Uusaro A., Lahtinen P. (2019). Vasoactive-inotropic score and the prediction of morbidity and mortality after cardiac surgery. Br. J. Anaesth..

[21] Garcia R.U., Walters H.L., Delius R., Aggarwal S. (2016). Vasoactive inotropic score (VIS) as biomarker of short-term outcomes in adolescents after cardiothoracic surgery. Pediatr. Cardiol..

[22] Adamson G.T., Arunamata A., Tacy T.A., Silverman N.H., Ma M., Maskatia S.A., Punn R. (2020). Postoperative recovery of left ventricular function following repair of large ventricular septal defects in infants. J. Am. Soc. Echocardiogr..

[23] Klitsie L.M., Kuipers I.M., Roest A.A.W., Van Der Hulst A.E., Stijnen T., Hazekamp M.G., Blom N.A., Harkel A.D.T. (2013). Disparity in right vs. left ventricular recovery during follow-up after ventricular septal defect correction in children. Eur. J. Cardio-Thorac. Surg..

[24] Klitsie L.M., Roest A.A.W., Kuipers I.M., Hazekamp M.G., Blom N.A., Ten Harkel A.D.J. (2014). Left and right ventricular performance after arterial switch operation. J. Thorac. Cardiovasc. Surg..

[25] Howard-Quijano K., Methangkool E., Scovotti J.C., Mazor E., Grogan T.R., Kratzert W.B., Mahajan A. (2019). Regional left ventricular myocardial dysfunction after cardiac surgery characterized by 3-dimensional strain. Anesth. Analg..

[26] Durmaz T., Bayram H., Bayram N., Sari C., Keles T., Bastug S., Bozkurt E. (2014). Effect of coronary artery bypass surgery on left ventricular function as assessed by strain and strain rate imaging. Perfusion.

